# Dissecting the Oncogenic Roles of Keratin 17 in the Hallmarks of Cancer

**DOI:** 10.1158/0008-5472.CAN-21-2522

**Published:** 2022-04-01

**Authors:** Gabriella Baraks, Robert Tseng, Chun-Hao Pan, Saumya Kasliwal, Cindy V. Leiton, Kenneth R. Shroyer, Luisa F. Escobar-Hoyos

**Affiliations:** 1 Undergraduate Program in Biomedical Engineering, Stony Brook University, Stony Brook, New York.; 2 Department of Pathology, Renaissance School of Medicine, Stony Brook University, Stony Brook, New York.; 3 Molecular and Cellular Biology Graduate Program, Stony Brook University, New York.; 4 Department of Therapeutic Radiology and Molecular Biophysics and Biochemistry, Yale University, New Haven, Connecticut.

## Abstract

There is an unmet need to identify and validate tumor-specific therapeutic targets to enable more effective treatments for cancer. Heterogeneity in patient clinical characteristics as well as biological and genetic features of tumors present major challenges for the optimization of therapeutic interventions, including the development of novel and more effective precision medicine. The expression of keratin 17 (K17) is a hallmark of the most aggressive forms of cancer across a wide range of anatomical sites and histological types. K17 correlates with shorter patient survival, predicts resistance to specific chemotherapeutic agents, and harbors functional domains that suggest it could be therapeutically targeted. Here, we explore the role of K17 in the hallmarks of cancer and summarize evidence to date for K17-mediated mechanisms involved in each hallmark, elucidating functional roles that warrant further investigation to guide the development of novel therapeutic strategies.

## Introduction

Tumor-specific features present the opportunity to develop precision medicine approaches by serving as biomarkers and therapeutic targets. While technologies have advanced the search for specific druggable candidates or molecular pathways, few have been successfully implemented to treat cancer. Ideal therapeutic targets include those that are specifically expressed in cancer cells or the tumor microenvironment, such as human epidermal growth factor receptor 2 (HER2) in breast cancer [targeted by Herceptin (trastuzumab)], and the epidermal growth factor receptor (EGFR) in lung and colorectal cancers [targeted by Erbitux (cetuximab) among other EGFR inhibitors; refs. 1–3]. Despite this progress, cancer remains the second leading cause of mortality in the United States (4) and in most industrialized nations, and other potentially targetable cancer biomarkers remain widely unexplored. Thus, identifying novel biomarkers that can be leveraged for the development of more effective therapeutic interventions is urgently needed.

**Figure 1.**
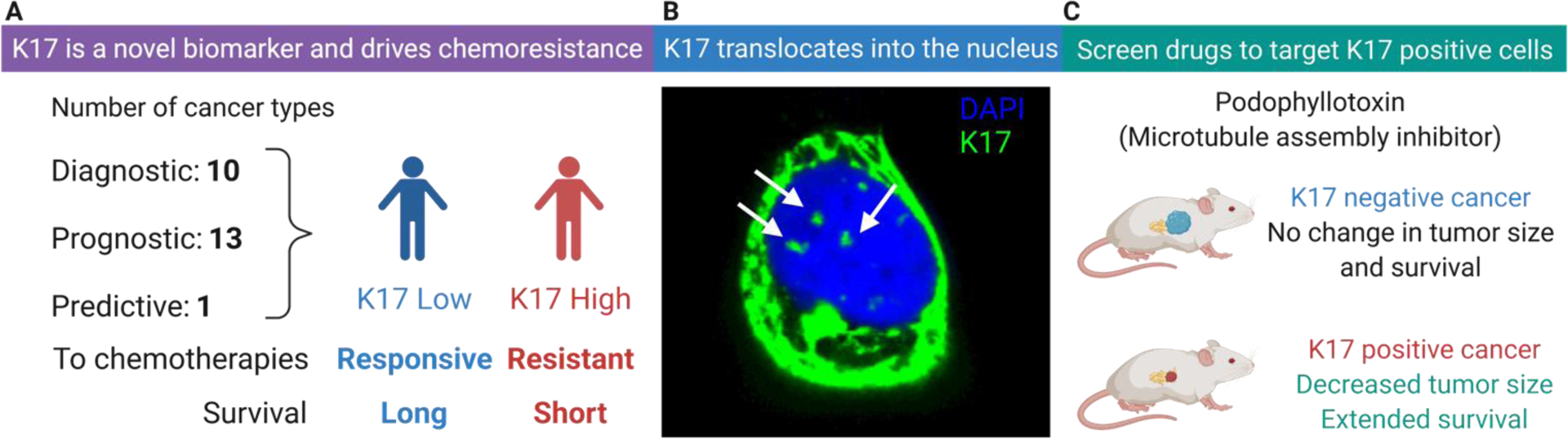
Significant findings on K17 suggest it is an ideal therapeutic target. **(A)** Overall, K17 is a predictive, prognostic, and diagnostic biomarker in several different cancers. K17 was previously found to predict therapeutic response of tumors, such that low K17 expression in tumors is correlated with longer patient survival and high K17 expression in tumors is correlated with shorter survival in patients. K17 was shown to promote chemoresistance to first-line chemotherapeutic regimens that do not target K17. **(B)** K17 translocates into the nuclei of cancer cells to promote tumorigenic functions. Confocal imaging shows K17 (green) and nucleus staining with DAPI (blue). **(C)** An unbiased high-throughput drug screen revealed several potential molecules that can target K17-expressing PDAC cells, including podophyllotoxin, a microtubule assembly inhibitor.

Over the past decade, concepts have emerged that histologically similar cancer cases can be highly diverse at the level of gene expression, and that these differences are highly significant and prognostically relevant. The classification of molecular subtypes based on gene expression signatures have been established for a wide range of cancers, including pancreatic (5), cervical (6), bladder (7), lung (8), breast (9), and colorectal cancers (10). These studies have suggested that some tumors that arise at different anatomic sites are highly similar at the transcriptome level. While the components of prognostic signatures have limited overlap across studies within a given cancer type, keratin 17 (K17) has been independently and consistently found to be a defining feature of the most aggressive subset of cancers across several anatomic sites. In cancer, K17 is reported to be expressed in at least 20 anatomic sites and based on the human protein atlas (https://www.proteinatlas.org), the expression of K17 is characterized as high/medium in colorectal, head and neck, stomach, pancreatic, urothelial, breast, cervical, skin, ovarian, lung, endometrial, carcinoid, and thyroid cancers. In contrast, the expression of K17 is characterized as low in prostate, liver, renal, melanoma, testis, glioma, and lymphoma cancers. The gradient of K17 expression depicts it as a promising and novel prognostic (11–23), diagnostic (24–33), and predictive biomarker (Fig. 1A; and Supplementary Table S1; ref. 34). Notably, K17 is a signature gene of the most aggressive form of basal-like subtype in pancreatic cancer (5). Furthermore, K17 has been developed as a noninvasive diagnostic test (URO17; ref. 35) to enhance the accuracy of initial diagnosis of urothelial carcinoma and to monitor for urothelial carcinoma recurrence following treatment (27). Importantly, the mechanistic roles of K17 have been investigated in a diverse range of cancer models (14, 17, 34, 36–45), where K17 has been found to impact multiple hallmarks of cancer (14, 36–38, 40, 44,46–49). Therefore, uncovering the mechanisms through which K17 promotes cancer aggression will guide future studies to explore K17 as a therapeutic target for the most aggressive forms of cancer (Fig. 1B and C).

**Figure 2.**
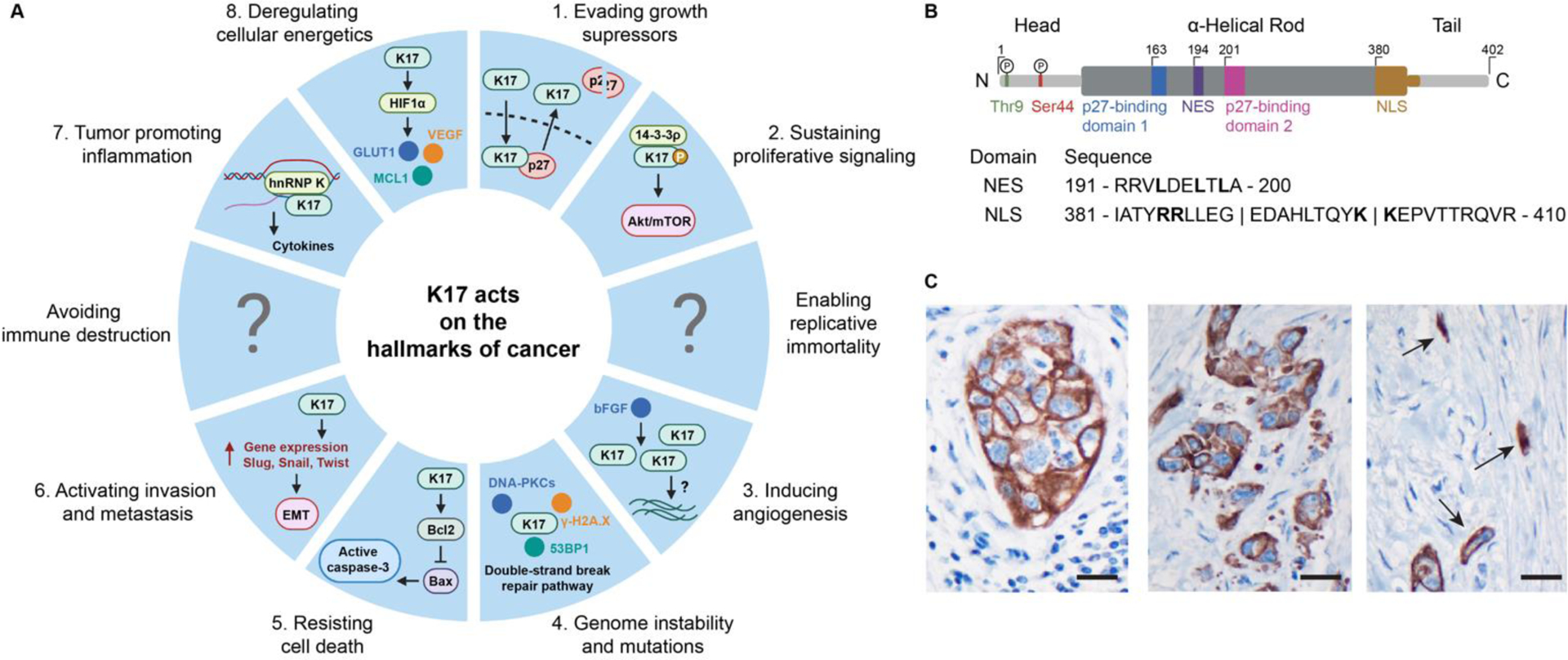
**(A) The implication of K17 in each of the 10 deadly hallmarks of cancer.** K17 has a function in several hallmarks of cancer. Each piece of the pie chart resembles a hallmark of cancer and represents the mechanism K17 is reported to have in this hallmark, as highlighted in blue. The regions that contain a question mark portray a lack of evidence for K17 in this hallmark and signify that further studies are needed to see whether K17 works mechanistically in cells in this feature. **(B) Structure of K17.** K17 is made up of an *α*-helical filament domain (residues 84–392) sectioned into 4 parts of repeated heptads (1A, 1B, 2A, and 2B), and non-helical head (N terminal; residues 1–83) and tail (C terminal; residues 393–432) domains. K17 has a Nuclear Export Signal (NES) found between residues 191 and 200 of the filament domain and a Nuclear Localization Signal (NLS) found between residues 381 and 410 of the protein. It has also been recently reported that there are 2 phosphorylation sites found on the N-terminal head domain of K17, serine 44 (Ser44) and threonine 9 (Thr9) **(C) Immunohistochemical localization of K17 in PDAC.** Note diverse patterns of stained tumor cells. Cohesive cluster of large tumor cells (left); Smaller tumor cells and apoptotic debris (middle); K17 highlighting small diffusely infiltrative tumor cells, embedded in a densely desmoplastic stroma (right). Original magnification 600x, scale bars 20 um.

Cancer cells exhibit a wide range of properties, known as the “hallmarks of cancer”, that allow them to thrive in inhospitable environments (50). Here, we summarize studies that have uncovered K17’s functions, domains, and “partners in crime” in these hallmarks (Fig. 2A). We comprehensively discuss the signaling pathways that are regulated by K17 and highlight future studies required to elucidate key aspects of the biology of K17 in cancer.

## K17 Is a Dynamic Functional Protein in Cancer

Keratins are a subtype of intermediate filament proteins, including 28 acidic type I proteins and 26 basic type II proteins. These cytoskeletal proteins have cellular and molecular functions involved in structural support, modulating several signaling pathways and metabolic processes and in maintaining cellular integrity (51, 52). K17 is a type I acidic intermediate filament composed of a highly structured α-helical rod and an intrinsically disordered non-helical head and tail (Fig. 2B; ref. 52). This intrinsically disordered state has thus far prevented the study of its complete crystal structure (53).

K17 is normally expressed during embryogenesis, silenced in mature somatic tissues except in certain stem cell populations (54, 55), and reexpressed in some cancers (Fig. 2C; refs. 27, 56). Mechanisms that drive K17 expression in cancer, however, have not yet been determined. In normal embryonic development, K17 functions in the onset of the development of placodes, the precursors that give rise to hair, glands, and teeth, and the morphogenesis of skin epithelia, where it regulates cell growth and motility (57, 58). In adult somatic tissues, K17 expression is also induced in response to stress, including tissue injury and in response to inflammation, as occurs in psoriasis (59) and in epithelial transition zones including the ocular limbus, the pectinate line of the anorectal mucosa, and the gastroesophageal junction (60). For example, a population of K17-positive basal cells with multipotent properties at the anorectal junction was found to be critical in maintaining squamous epithelium during normal homeostasis and repairing glandular epithelium following tissue injury (61). K17’s expression in transition zones and its role in progressing malignancy in those areas warrants further investigation.

K17 has been found to harbor functional domains that enable it to bind to other functional proteins (62), translocate to cellular compartments (17), including the nucleus, and undergo post-translational modifications (63). It is unknown, however, how keratin filament dynamics are regulated and their function in cancer. Posttranslational modifications of the domains and sites of K17, including phosphorylation, acetylation, or ubiquitylation, have been linked to regulation of cellular functions, but have not yet been fully characterized (Fig. 2B). Yang and colleagues (64) used mass spectrometry and immunohistochemistry to demonstrate that K17 can bind and colocalize with E3 ubiquitin-ligase and the tripartite motif-containing protein 21 (Trim21) in HaCaT cells (an immortalized keratinocyte cell line widely used as a model of psoriasis). Furthermore, Trim21 was determined to drive the ubiquitination of K17 via lysine-63–linked polyubiquitin chains. This stabilized the expression of K17, resulting in the activation and nuclear translocation of signal transducers and activators of transcription 3 (STAT3), ultimately promoting cell proliferation in psoriatic cells. Thus, discovering post-translational modifications of K17 and identifying prospective binding partners and domains may also facilitate a better understanding of the mechanisms through which K17 impacts cell proliferation in cancer.

Beyond serving as a cytoskeletal protein, K17 can solubilize from the filamentous form and was the first keratin discovered to translocate into the nucleus via a canonical bipartite nuclear localization signal (NLS) and exit the nucleus by a nuclear export signal (NES), where it impacts multiple cellular properties in cancer cells (Figs. 1B and 2B; refs. 65, 66). We previously reported that soluble nuclear K17 functions in regulating the subcellular localization and degradation of p27^KIP1^ (p27) in cervical cancer (17). In addition, K17 regulates the expression of various transcription factors that have been found to underlie the pathogenesis of cutaneous basal cell carcinoma, including Aire and the heterogeneous nuclear ribonucleoprotein K (hnRNPK; ref. 44). Soluble nuclear K17 also impacts nuclear morphology and gene expression through its ability to impact chromatin organization (67), although the detailed mechanisms through which K17 regulates gene expression have not yet been fully dissected. Thus, across several cancers, K17 has been found to interact with key proteins inside and outside the nucleus, ultimately impacting cancer progression.

## K17 Targets Tumor Suppressors

Tumor suppressors are vital cell-cycle regulators, and their dysregulation contributes to tumorigenesis by promoting uncontrolled cell-cycle progression. K17 modulates cell-cycle progression by regulating the tumor suppressor p27 with its nuclear shuttling function by a classic and conserved NLS and NES. These signals enable the soluble nuclear K17 to serve as a nuclear shuttle of p27 (Fig. 2A, 1; ref. 17), and potentially, other tumor suppressor proteins. In cervical cancer, the nuclear export of p27 results in sustained cell-cycle progression and inadequate DNA replication, promoting cancer pathogenesis (17, 68). Moreover, K17 serves as a bridge between p27 and Exportin 1 (chromosomal maintenance 1, CRM1), a transport protein that has been implicated in the export of several tumor suppressors, including adenomatous polyposis coli (APC), p53, and breast cancer gene 1 (BRCA1; ref. 69). Further investigation is warranted to determine if K17 and CRM1 work together to export other tumor suppressors to regulate cancer progression. Importantly, a CRM1 inhibitor, Selinexor, has been tested in a clinical trial (NCT02178436) in combination with gemcitabine and paclitaxel as an effective therapy to treat metastatic pancreatic cancer (70). It is not yet known how CRM1 inhibition impacts K17’s ability to function as a nuclear shuttle, and whether this will be a potential therapeutic target for K17-positive cancers.

K17 has been implicated in connection to other critical tumor suppressors, such as BRCA1 and p53. In breast cancer cell lines, an inverse relationship was determined between BRCA1 and K17 transcription, such that K17 expression was repressed by functional BRCA1, and BRCA1 knockout resulted in an approximately a 6-fold increase in K17 (71). This may thereby explain the observed overexpression of key basal markers, including K17, in BRCA1-deficient breast tumors. Similarly, Liao and colleagues (72) found a negative correlation between K17 and p53 expression using a rat model of radiation dermatitis and concluded that p53 is a direct repressor of K17 transcription, which could provide a rationale for therapeutically targeting p53 in dermatoses.

Silencing K17 has been shown to lead to G_1_–S phase cell-cycle arrest in cervical cancer cell lines and tumor tissue from patients with gastric cancer (17, 46). Specifically in gastric cancer, this was due to decreased expression of cyclin E and D1 (46), supporting the conclusion that K17 regulates cell-cycle progression. Notably, silencing K17 caused an increase in the expression of tumor suppressors that mediate apoptosis, including Bcl-2-associated x protein (Bax) and cleaved caspase-3 (46). Taken together, these studies suggest that K17 can directly and indirectly target tumor suppressors.

## K17-Mediated Signaling Enhances Cell Proliferation and Tumor Growth

Cancer cells are immortal, enabling unlimited replicative capacity typically driven by upregulation of telomerase to stabilize telomeric DNA and the deregulation of specific signaling pathways (73–76). Here, we discuss how K17 activates cell signaling in normal and cancerous cells, resulting in increased proliferation and cell size (Fig. 2A, 2).

Based on observations that K17-null keratinocytes were much smaller than those that expressed K17, a study concluded that K17 increases the mass and size of keratinocytes during skin development (47). The group further explored the effects of K17 on protein synthesis in the protein kinase B (Akt)/mammalian target of rapamycin (mTOR) pathway, which impacts cell proliferation, metabolism, angiogenesis, epithelial–mesenchymal transition (EMT), and invasion (77). They found that K17 acts downstream of phosphoinositide 3-kinase (PI3K) activation in the Akt/mTOR pathway, prompting protein synthesis and cell growth (47). Similarly, K17 was found to promote cellular proliferation and tumor growth in esophageal squamous cell carcinoma (ESCC). Khanom and colleagues (78) found that K17-mediated signaling stimulated the Akt/mTOR pathway, contributing to oral cancer cell line progression by promoting cell migration and proliferation. In ESCC animal models, K17-overexpressing cells implanted into mice resulted in larger tumors while K17-knockout attenuated tumor growth (36). Mouse tail vein injections supported the conclusion that K17 promotes cell migration and pulmonary metastasis, which are accompanied by the activation of Akt signaling (36). In bladder cancer cell lines, K17 was found to be upregulated, and its knockdown resulted in the suppression of colony formation and invasion (79). Specifically, K17 regulated the expression of EMT markers such that silencing K17 resulted in increased E-cadherin and decreased N-cadherin and correlated with suppressed invasion and metastasis. Silencing K17 decreased the expression of oncogenic phosphorylated-ERK and phosphorylated–Akt signaling (79).

Furthermore, K17 contributes to the pathogenesis of both Ewing sarcoma and basal cell carcinoma (BCC) through glioma-associated oncogene (Gli) proteins, which are transcriptional effectors of the sonic hedgehog (SHH) pathway. In Ewing’s sarcoma, K17 was found to induce Akt signaling, to mediate cellular adhesion (37), and upregulate GLI1, both to activate and repress cell–cell adhesion. In BCC, a cancer in which K17 is found to be highly upregulated (26), SHH signaling, which promotes cell proliferation, is upregulated through the activation of Gli protein expression (80). Callahan and colleagues (81) evaluated the promoter activity of K17 in BCC to determine whether the actin binding protein, Missing in Metastasis (MIM), is an effective SHH responsive gene and can affect Gli transcriptional outputs. They ultimately implicated K17 as a direct target gene of Gli such that Gli1 and Gli2 can induce K17 expression. It would be interesting to further see if there is a direct mechanistic link between K17 and SHH signaling as they both share a relationship with transcription factors of Gli.

K17 affects oncogenic signaling pathways, contributing to its effect on cancer progression. Chung and colleagues (39) used a epidermoid carcinoma cell line to illustrate that K17 binds to and facilitates the expression, phosphorylation, and subcellular localization of Annexin A2 (AnxA2) in response to EGFR activation. Furthermore, 14–3–3σ, a negative cell-cycle regulator, was identified as a K17-binding protein, depending on the phosphorylation of serine 44 (S44) and threonine 9 (T9) of the nonhelical head of K17 (Fig. 2B; ref. 82). Binding of 14–3–3σ to S44 and T9 phosphorylation sites of K17 promoted nuclear export of 14–3–3σ and cell growth in skin keratinocytes (47). In cell lines derived from oral carcinoma *in situ* and SCC, immunohistochemistry revealed that K17 and the tumor suppressor 14–3–3σ are coexpressed (38). K17 knockdown resulted in significantly decreased cell number and slowed cell migration. Although K17 binding to 14–3–3σ lead to nuclear export of 14–3–3σ, the underlying mechanisms that mediate this process are unknown. Specifically, it is not known if K17–14–3–3σ export is mediated by CRM1 or another transport protein. Enaka and colleagues (83) explored the role of K17 in promoting proliferation and invasion in oral SCC by investigating the relationship between K17 and p53 mutations and reported that overexpressing mutant p53 (p53^R248W^) resulted in the suppression of K17 expression, reducing activity in proliferation, cell size, and invasion.

The observations that K17 mediates the nuclear export of 14–3–3σ, p27 and p53, suggests that there could be common underlying mechanism through which K17 has a generalizable role as a nuclear shuttle of tumor suppressor proteins to drive cell cycle progression and tumor growth. Targeting these associated pathways could be a therapeutic strategy to inhibit K17-expressing cancer cell growth.

## K17 in Angiogenesis

Although K17 has not been shown to directly regulate angiogenesis, its immunomodulatory role has been reported to be coincide with angiogenesis. K17 knockout correlates with decreased vascularization of cutaneous basal cell carcinomas, as a result of decreased expression of platelet endothelial cell adhesion molecule-1 (PECAM-1 or CD31), a modulator of angiogenesis and endothelial cell migration (Fig. 2A, 3; ref. 40). Although Xu and colleagues (41) concluded that K17 does not directly modulate angiogenesis, it was postulated that the induction of angiogenic factors elevated K17 expression, resulting in endothelial tube formation *in vitro*. Of note, other keratins, including keratin 19 (84) and keratin 14 (85) have been linked to the regulation of angiogenesis in hepatocellular carcinoma and cervical squamous cell carcinoma, respectively. Therefore, the role of K17 in the regulation of this hallmark of cancer is relatively unexplored and should be further interrogated.

## K17 in DNA Damage Response

DNA damage response involves numerous signaling events, including the regulation of the cell cycle and DNA replication, and is fundamentally important to genomic instability as it impacts tumorigenesis. Nair and colleagues (42) reported that nuclear K17 is induced in response to DNA damage and that K17 is required in the early double-strand break (DSB) repair pathway in tumor keratinocytes (Fig. 2A, 4). Nuclear K17 immunoprecipitated with key proteins of the DSB repair pathway, including γH2AX, 53BP1, and DNA-PKCs (42). A DNA damage response involving K17 allowed keratinocytes to survive after DNA damage, while cells with K17 knockout or lack of nuclear-K17 showed decreased survival due to dysregulation of the DSB pathway (42). Because the loss of K17 dysregulates DSB repair, pharmacologic inhibition of K17 could promote genomic instability, creating vulnerabilities that could be therapeutically manipulated. Although the paradigm of synthetic lethality in vulnerable pathways has been established (i.e., PARP inhibitors in patients with BRCA1/BRCA2 mutations; ref. 86), it is unknown if targeting K17 could destabilize DNA repair pathways as a synthetic lethality approach in a similar fashion.

## K17 Mediates Resistance to Programmed Cell Death and Chemoresponse

K17 regulates apoptotic cell death in the pathogenesis of multiple types of cancer (Figs. 2A, 5 and 1D). Hu and colleagues (46) observed that silencing K17 decreased the expression of Bcl2 and cleaved caspase-3, and thereby induced apoptosis in human gastric carcinoma tissues. Furthermore, Tong and colleagues (87) found that K17 modulates TNFα signaling by interacting with TNF receptor 1 (TNFR1)-associated death domain protein (TRADD). They further concluded that TNFα signaling was enhanced in K17-null mouse skin tissue, as measured by increased NF-κB activity (82).

Chemoresistance is an important barrier for the development of novel and more effective approaches to treat cancer. Our group reported that K17 expression drives a greater than two-fold increase, both *in vitro* and *in vivo*, in resistance to gemcitabine and 5-fluorouracil, commonly used as first line agents to treat pancreatic ductal adenocarcinoma (PDAC; ref. 34). Similarly, we found that the sensitivity to cisplatin, a first-line therapeutic agent for cervical cancer, was increased two-fold by K17 knockdown (17). The resistance to cisplatin induced by K17 was later confirmed in bladder cancer cells (79). In addition, Li and colleagues (43) reported that K17 drives chemoresistance to paclitaxel in cervical cancer cells (43).

To explore opportunities to overcome chemoresistance in K17-positive cancers, our team performed an unbiased high-throughput screen in pancreatic cancer models (34) and found that podophyllotoxin, a microtubule assembly inhibitor, was at least two-fold more potent in K17-positive compared to K17-negative PDAC cells (Fig. 1C). Another microtubule disassembly inhibitor, paclitaxel, is currently used in combination with gemcitabine as a first line chemotherapeutic regimen for PDAC, breast, lung, and ovarian cancer. Surprisingly, we found that when combined with gemcitabine, podophyllotoxin but not paclitaxel, showed strong synergistic effects in inhibiting the viability of K17-expressing PDAC cells. Thus, these experiments suggest that targeting microtubule assembly rather than microtubule disassembly is a therapeutic opportunity in K17-expressing pancreatic cancers and that podophyllotoxin and its chemotherapeutic derivatives, such as etoposide, could potentially be combined with gemcitabine to enhance treatment efficacy for K17-positive PDACs.

Taken together, these observations demonstrate that K17 mediates resistance to apoptosis and drives chemoresistance; thus, targeting K17 could be a strategy to enhance therapeutic efficacy. Further studies are indicated to address the underlying mechanisms of resistance and to leverage this information to design more effective therapies for K17-expressing cancers.

## K17 Regulates Migration and Invasion

K17 interacts with Akt, a mediator of EMT and cell migration in cancer (Fig. 2A, 6). However, the role of K17 has been reported inconsistently in multiple studies. In esophageal squamous cell carcinoma, transcription factors that drive EMT, including Slug, Snail, and Twist increase in response to upregulation of K17 expression, but decrease in K17-knockout cells (36). Chiang and colleagues (48) reported that cells that express K17 promote EMT in oral squamous cell carcinoma. Similarly, in non-small cell lung cancer, elevated levels of K17 promote cell proliferation, colony formation, and invasion, while down-regulation of K17 has opposite effects (14). Hu and colleagues (46) also reported that downregulation of K17 suppressed proliferation and migration *in vitro* and reduced tumorigenicity and invasion *in vivo* in gastric cancer models. In PDAC, pancreatic stellate cells secrete TGFβ1, which negatively regulates L1 cell adhesion molecule (L1CAM) expression, resulting in a more aggressive PDAC phenotype (88). Importantly, in low L1CAM-expressing tumors, there is increased expression of K17. This is in line with the concept that K17 advances tumor aggression through promoting stemness and decreasing cell adhesion by a potential interaction with L1CAM.

In contrast, Zeng and colleagues (89) reported that knocking down K17 expression resulted in a significant decrease of E-cadherin and promoted the expression of vimentin in pancreatic cancer cell lines. Through cell proliferation and colony formation assays *in vitro*, K17 significantly inhibited cell proliferation. K17 also suppressed migration and invasion as found through wound healing and transwell invasion experiments. Overall, these results paradoxically indicate K17 as a tumor suppressor in their model systems. Consistent with their findings, Quinn and colleagues (90) reported that K17 was found to be negatively correlated with metastatic phenotype in lung cancer xenografts. Thus, data on the impact of K17 on invasion and migration are inconclusive, potentially due to phenotypic differences between cancer models. The relationship between K17 and EMT warrants further investigation to clarify these inconsistencies.

## K17 Is Involved in the Immune Regulatory Network

In cutaneous BCC, the overexpression of K17 promotes tumorigenesis and impacts the inflammatory microenvironment (Fig. 2A, 7; ref. 91). K17 levels directly correlate with changes in the expression of inflammatory T-helper cytokines in BCCs, including Th1, Th2, and Th17 (40). Additionally, K17 impacts tumor promoter TPA (12-O-tetradecanoylphorbol-13-acetate)-induced expression of certain chemokines, including *Cxcl11*, *Cxcl5*, *Cxcl9 and Cxcl10* in TPA-treated skin keratinocytes (27, 31). In a mouse papillomavirus model with induced K17, the expression of *Cxcl9* and *Cxcl10* were inhibited, resulting in decreased infiltration of CD8^+^ T cells (92). This suggests that K17 could block T-cell infiltration, and thereby impact the inflammatory microenvironment in cancer.

Hobbs and colleagues has reported that nuclear K17 regulates the expression of an autoimmune regulator, Aire, by interacting with other factors, including hnRNP K. In addition, p65 (NF-kB) has been reported to be a potential molecular bridge between K17 and Aire, resulting in increased proinflammatory gene expression and tumor growth (44). Furthermore, Lo and colleagues used immunohistochemistry and illustrated that K17 colocalized with key cytokines, including *Cxcr3, Cxcl10*, and *Cxc11* in BCC (93).

The involvement of K17 in immune changes has also been examined in human papillomavirus (HPV) type 16 mouse models of cervical dysplasia (49). Lesions that were K17-positive had a two-fold increase in the level of transcripts involved in signaling and growth pathways including Notch and Wnt, and the transcript levels for pro-inflammatory cytokines were significantly elevated, including *Ifng, Cxcl9, Cxc110, Cxc111, Ido1, Mmp13 Tnfa, I11b, Mmp9, Tgfb*, and *Cxc15* (49). Although K17 expression has been found to induce tumor-promoting inflammation in BCC (40, 44) and cervical SCC (49), the potential interactions between K17 and the inflammatory microenvironment have not yet been explored in other cancer types. Thus, K17 impacts the immune microenvironment at multiple levels, but further studies are indicated to uncover the interactions between tumor cells relative to K17-status, and tumor-associated cytotoxic T cells, pro-tumor (M2) versus anti-tumor (M1) macrophages, and other mediators of the immune response, to determine if targeting K17 expression could enable more effective immunotherapeutic approaches for cancer.

## K17 Alters Metabolism

Cancer cells have the ability to fundamentally reprogram pathways for energy production to enable a switch from aerobic glycolysis to anaerobic metabolism, otherwise known as the Warburg effect (50). Anaerobic metabolism is associated with chemoresistance and can be therapeutically targeted (94, 95). K17 impacts cancer cell metabolism in osteosarcoma via the Akt/mTOR/hypoxia-inducible factor (HIF)-1α pathway (96). Furthermore, mRNA and protein expression levels of target genes of HIF1α, including *GLUT1*, *MCL1*, and *VEGF*, decreased in response to K17 inhibition (96), and K17 knockdown in osteosarcoma induced G1 arrest and inhibited glycolysis *in vitro* (Fig.2A, 8). While these observations are intriguing, further studies are needed to better understand how K17 alters cancer metabolism and whether this hallmark of K17 expression contributes to chemoresistance.

## Conclusions

This review summarizes and highlights studies that have addressed the interactions of K17 with several hallmarks of cancer, establishing it as a potential therapeutic target. K17 impacts many proteins and pathways that drive biologic aggression, including the promotion of sustained cell-cycle progression, invasion, and angiogenesis, reprograming the metabolome, and chemoresistance. The reexpression of this embryonic keratin in cancer, its association to the most aggressive molecular subtypes of carcinomas across anatomic sites, and its mechanistic links in multiple hallmarks of cancer emphasize the importance of this protein for tumorigenesis and tumor maintenance. Thus, K17 may represent an opportunity to treat the most aggressive subtypes of cancer, based on biomarkers of gene expression rather than mutation status.

## Supplementary Material

Supplmental Materials

## References

[1] Antonicelli A, Cafarotti S, Indini A, Galli A, Russo A, Cesario A, EGFR-targeted therapy for non-small cell lung cancer: focus on EGFR oncogenic mutation. Int J Med Sci 2013;10:320–30. 23423768 10.7150/ijms.4609 PMC3575628

[2] Vecchione L, Jacobs B, Normanno N, Ciardiello F, Tejpar S. EGFR-targeted therapy. Exp Cell Res 2011;317:2765–71. 21925171 10.1016/j.yexcr.2011.08.021

[3] Nielsen DL, Andersson M, Kamby C. HER2-targeted therapy in breast cancer. Monoclonal antibodies and tyrosine kinase inhibitors. Cancer Treat Rev 2009; 35:121–36. 19008049 10.1016/j.ctrv.2008.09.003

[4] Centers for Disease Control and Prevention, N.C.f.H.S. Underlying Cause of Death 1999–2019 on CDC WONDER Online Database. 2020.

[5] Moffitt RA, Marayati R, Flate EL, Volmar KE, Loeza SGH, Hoadley KA, Virtual microdissection identifies distinct tumor- and stroma-specific subtypes of pancreatic ductal adenocarcinoma. Nat Genet 2015;47:1168–78. 26343385 10.1038/ng.3398 PMC4912058

[6] Cancer Genome Atlas Research, N. Albert Einstein College of Medicine, Analytical Biological Services, Barretos Cancer Hospital, Baylor College of Medicine, Beckman Research Institute of City of Hope, Buck Institute for Research on Aging, Integrated genomic and molecular characterization of cervical cancer. Nature 2017;543:378–84. 28112728 10.1038/nature21386 PMC5354998

[7] Fong MHY, Feng M, McConkey DJ, Choi W. Update on bladder cancer molecular subtypes. Translational andrology and urology 2020;9:2881–9. 33457262 10.21037/tau-2019-mibc-12 PMC7807369

[8] Hu F, Zhou Y, Wang Q, Yang Z, Shi Y, Chi Q, Gene Expression Classification of Lung Adenocarcinoma into Molecular Subtypes. IEEE/ACM Trans Comput Biol Bioinform 2020;17:1187–97. 30892233 10.1109/TCBB.2019.2905553

[9] Fragomeni SM, Sciallis A, Jeruss JS. Molecular subtypes and local-regional control of breast cancer. Surg Oncol Clin N Am 2018;27:95–120. 29132568 10.1016/j.soc.2017.08.005 PMC5715810

[10] Guinney J, Dienstmann R, Wang X, de Reyni es A, Schlicker A, Soneson C, The consensus molecular subtypes of colorectal cancer. Nat Med 2015;21: 1350–6. 26457759 10.1038/nm.3967 PMC4636487

[11] Kim K, Lee HW, Chae SW, Kim DH, Do IG, Lee HJ, Cytokeratin 17 expression is associated with poor prognosis in gallbladder adenocarcinoma. Appl Immunohistochem Molecul Morphol 2017;25:346–50. 10.1097/PAI.0000000000000307 26990743

[12] Ide M, Kato T, Ogata K, Mochiki E, Kuwano H, Oyama T. Keratin 17 expression correlates with tumor progression and poor prognosis in gastric adenocarcinoma. Ann Surg Oncol 2012;19:3506–14. 22695933 10.1245/s10434-012-2437-9

[13] Wang Y-F, Lang H-Y, Yuan J, Wang J, Wang R, Zhang X-H, Overexpression of keratin 17 is associated with poor prognosis in epithelial ovarian cancer. Tumour Biol 2013;34:1685–9. 23430585 10.1007/s13277-013-0703-5

[14] Wang Z, Yang M-Q, Lei L, Fei L-R, Zheng Y-W, Huang W-J, Overexpression of KRT17 promotes proliferation and invasion of non-small cell lung cancer and indicates poor prognosis. Cancer Manag Res 2019;11:7485–97. 31496806 10.2147/CMAR.S218926 PMC6689799

[15] Yagyuu T, Obayashi C, Ueyama Y, Takano M, Tanaka Y, Kawaguchi M, Multivariate analyses of Ki-67, cytokeratin 13 and cytokeratin 17 in diagnosis and prognosis of oral precancerous lesions. J Oral Pathol Med 2015;44:523–31. 25243470 10.1111/jop.12262

[16] Liu ZB, Wu J, Ping B, Feng LQ, Shen ZZ, Shao ZM. [Expression of CK5/6 and CK17 and its correlation with prognosis of triple-negative breast cancer patients]. Zhonghua Zhong Liu Za Zhi 2008;30:610–4. 19102940

[17] Escobar-Hoyos LF, Shah R, Roa-Peña L, Vanner EA, Najafian N, Banach A, Keratin-17 promotes p27KIP1 Nuclear export and degradation and offers potential prognostic utility. Cancer Res 2015;75:3650–62. 26109559 10.1158/0008-5472.CAN-15-0293

[18] Merkin RD, Vanner EA, Romeiser JL, Shroyer ALW, Escobar-Hoyos LF, Li J, Keratin 17 is overexpressed and predicts poor survival in estrogen receptornegative/human epidermal growth factor receptor-2-negative breast cancer. Hum Pathol 2017;62:23–32. 27816721 10.1016/j.humpath.2016.10.006

[19] Mockler D, Escobar-Hoyos LF, Akalin A, Romeiser J, Shroyer AL, Shroyer KR. Keratin 17 Is a Prognostic Biomarker in Endocervical Glandular Neoplasia. Am J Clin Pathol 2017;148:264–73. 28821199 10.1093/ajcp/aqx077

[20] Regenbogen E, Mo M, Shroyer ALW. Escobar-Hoyos LF, Burke S. Elevated expression of keratin 17 in oropharyngeal squamous cell carcinoma is associated with decreased survival. Head Neck 2018;40:1788–98. 29626364 10.1002/hed.25164

[21] Roa-Peña L, Leiton CV, Babu S, Pan C-H, Vanner EA, Akalin A, Keratin 17 identifies the most lethal molecular subtype of pancreatic cancer. Sci Rep 2019;9:11239. 31375762 10.1038/s41598-019-47519-4 PMC6677817

[22] Bai JDK, Babu S, Roa-Peña L, Hou W, Akalin A, Escobar-Hoyos LF, Keratin 17 is a negative prognostic biomarker in high-grade endometrial carcinomas. Hum Pathol 2019;94:40–50. 31655172 10.1016/j.humpath.2019.09.005

[23] Roa-Pena L, Babu S, Leiton CV, Wu M, Taboada S, Akalin A, Keratin 17 testing in pancreatic cancer needle aspiration biopsies predicts survival. Cancer Cytopathol 2021;129:865–73. 34076963 10.1002/cncy.22438 PMC9014629

[24] Dasgupta S, Ewing-Graham PC, van Kemenade FJ, van Doorn HC, Hegt VN, Koljenović S, Differentiated vulvar intraepithelial neoplasia (dVIN): the most helpful histological features and the utility of cytokeratins 13 and 17. Archiv für Pathologische Anatomie und Physiologie und für Klinische Medicin 2018; 473:739–47. 10.1007/s00428-018-2436-8 PMC6267258 30187167

[25] Escobar-Hoyos LF, Yang J, Zhu J, Cavallo J-A, Zhai H, Burke S, Keratin 17 in premalignant and malignant squamous lesions of the cervix: proteomic discovery and immunohistochemical validation as a diagnostic and prognostic biomarker. Mod Pathol 2014;27:621–30. 24051697 10.1038/modpathol.2013.166 PMC4026928

[26] Anderson-Dockter H, Clark T, Iwamoto S, Lu M, Fiore D, Falanga JK, Diagnostic utility of cytokeratin 17 immunostaining in morpheaform basal cell carcinoma and for facilitating the detection of tumor cells at the surgical margins. Dermatol Surg 2012;38:1357–66. 22691048 10.1111/j.1524-4725.2012.02417.x PMC3412942

[27] Babu S, Mockler DC, Roa-Peña L, Szygalowicz A, Kim NW, Jahanfard S, Keratin 17 is a sensitive and specific biomarker of urothelial neoplasia. Mod Pathol 2019;32:717–24. 30443013 10.1038/s41379-018-0177-5

[28] Kitamura R, Toyoshima T, Tanaka H, Kawano S, Kiyosue T, Matsubara R, Association of cytokeratin 17 expression with differentiation in oral squamous cell carcinoma. J Cancer Res Clin Oncol 2012;138:1299–310. 22466643 10.1007/s00432-012-1202-6 PMC3397222

[29] Cohen-Kerem R, Rahat MA, Madah W, Greenberg E, Sabo E, Elmalah I. Cytokeratin-17 as a potential marker for squamous cell carcinoma of the larynx. Ann Otol Rhinol Laryngol 2004;113:821–7. 15535145 10.1177/000348940411301008

[30] Lok T, Chen L, Lin F, Wang HL. Immunohistochemical distinction between intrahepatic cholangiocarcinoma and pancreatic ductal adenocarcinoma. Hum Pathol 2014;45:394–400. 24439226 10.1016/j.humpath.2013.10.004

[31] Nazarian RM, Primiani A, Doyle LA, Linskey KR, Duncan LM, Odze RD, Cytokeratin 17: an adjunctive marker of invasion in squamous neoplastic lesions of the anus. Am J Surg Pathol 2014;38:78–85. 24335642 10.1097/PAS.0000000000000111

[32] Chen Y, Cui T, Yang L, Mireskandari M, Knoesel T, Zhang Q, The diagnostic value of cytokeratin 5/6, 14, 17, and 18 expression in human non-small cell lung cancer. Oncology 2011;80:333–40. 21791943 10.1159/000329098

[33] Kim CY, Jung WY, Lee HJ, Kim HK, Kim A, Shin BK. Proteomic analysis reveals overexpression of moesin and cytokeratin 17 proteins in colorectal carcinoma. Oncol Rep 2012;27:608–20. 22076435 10.3892/or.2011.1545

[34] Pan C-H, Otsuka Y, Sridharan B, Woo M, Leiton CV, Babu S, An unbiased high-throughput drug screen reveals a potential therapeutic vulnerability in the most lethal molecular subtype of pancreatic cancer. Mol Oncol 2020;14:1800–16. 32533886 10.1002/1878-0261.12743 PMC7400780

[35] Cucci J A better future for bladder cancer. The Pathologist 2019.

[36] Liu Z, Yu S, Ye S, Shen Z, Gao L, Han Z, Keratin 17 activates AKT signalling and induces epithelial-mesenchymal transition in oesophageal squamous cell carcinoma. J Proteomics 2020;211:103557. 31669361 10.1016/j.jprot.2019.103557

[37] Sankar S, Tanner JM, Bell R, Chaturvedi A, Randall RL, Beckerle MC, A novel role for keratin 17 in coordinating oncogenic transformation and cellular adhesion in Ewing sarcoma. Mol Cell Biol 2013;33:4448–60. 24043308 10.1128/MCB.00241-13 PMC3838177

[38] Mikami T, Maruyama S, Abé T, Kobayashi T, Yamazaki M, Funayama A, Keratin 17 is co-expressed with 14–3–3 sigma in oral carcinoma in situ and squamous cell carcinoma and modulates cell proliferation and size but not cell migration. Virchows Arch 2015;466:559–69. 25736868 10.1007/s00428-015-1735-6

[39] Chung B-M, Murray CI, Van Eyk JE, Coulombe PA. Identification of novel interaction between annexin A2 and keratin 17: evidence for reciprocal regulation. J Biol Chem 2012;287:7573–81. 22235123 10.1074/jbc.M111.301549 PMC3293598

[40] DePianto D, Kerns ML, Dlugosz AA, Coulombe PA. Keratin 17 promotes epithelial proliferation and tumor growth by polarizing the immune response in skin. Nat Genet 2010;42:910–4. 20871598 10.1038/ng.665 PMC2947596

[41] Xu Y, Zhang S-Z, Huang C-H, Liu X-Y, Zhong Z-H, Hou W-L, Keratin 17 identified by proteomic analysis may be involved in tumor angiogenesis. BMB Rep 2009;42:344–9. 19558792 10.5483/bmbrep.2009.42.6.344

[42] Nair RR, Hsu J, Jacob JT, Pineda CM, Hobbs RP, Coulombe PA. A role for keratin 17 during DNA damage response and tumor initiation. Proc Natl Acad Sci U S A 2021;118:e2020150118. 33762306 10.1073/pnas.2020150118 PMC8020757

[43] Li J, Chen Q, Deng Z, Chen X, Liu H, Tao Y, KRT17 confers paclitaxel-induced resistance and migration to cervical cancer cells. Life Sci 2019;224: 255–62. 30928404 10.1016/j.lfs.2019.03.065

[44] Hobbs RP, DePianto DJ, Jacob JT, Han MC, Chung B-M, Batazzi AS, Keratin-dependent regulation of Aire and gene expression in skin tumor keratinocytes. Nat Genet 2015;47:933–8. 26168014 10.1038/ng.3355 PMC4520766

[45] Liu J, Liu L, Cao L, Wen Q. Keratin 17 promotes lung adenocarcinoma progression by enhancing cell proliferation and invasion. Med Sci Monit 2018;24:4782–90. 29991674 10.12659/MSM.909350 PMC6069497

[46] Hu H, Xu D-H, Huang X-X, Zhu C-C, Xu J, Zhang Z-Z, Keratin17 Promotes Tumor Growth and is Associated with Poor Prognosis in Gastric Cancer. J Cancer 2018;9:346–57. 29344281 10.7150/jca.19838 PMC5771342

[47] Kim S, Wong P, Coulombe PA. A keratin cytoskeletal protein regulates protein synthesis and epithelial cell growth. Nature 2006;441:362–5. 16710422 10.1038/nature04659

[48] Chiang C-H, Wu C-C, Lee L-Y, Li Y-C, Liu H-P, Hsu C-W, Proteomics analysis reveals involvement of Krt17 in areca nut-induced oral carcinogenesis. J Proteome Res 2016;15:2981–7. 27432155 10.1021/acs.jproteome.6b00138

[49] Hobbs RP, Batazzi AS, Han MC, Coulombe PA. Loss of Keratin 17 induces tissue-specific cytokine polarization and cellular differentiation in HPV16-driven cervical tumorigenesis in vivo. Oncogene 2016;35:5653–62. 27065324 10.1038/onc.2016.102 PMC5333940

[50] Hanahan D, Weinberg RA. Hallmarks of cancer: the next generation. Cell 2011; 144:646–74. 21376230 10.1016/j.cell.2011.02.013

[51] Pan X, Hobbs RP, Coulombe PA. The expanding significance of keratin intermediate filaments in normal and diseased epithelia. Curr Opin Cell Biol 2013;25:47–56. 23270662 10.1016/j.ceb.2012.10.018 PMC3578078

[52] Jacob JT, Coulombe PA, Kwan R, Omary MB. Types I and II Keratin Intermediate Filaments. Cold Spring Harb Perspect Biol 2018;10:a018275. 29610398 10.1101/cshperspect.a018275 PMC5880164

[53] Lee C-H, Kim M-S, Chung BM, Leahy DJ, Coulombe PA. Structural basis for heteromeric assembly and perinuclear organization of keratin filaments. Nat Struct Mol Biol 2012;19:707–15. 22705788 10.1038/nsmb.2330 PMC3864793

[54] Moll R, Franke WW, Schiller DL, Geiger B, Krepler R. The catalog of human cytokeratins: patterns of expression in normal epithelia, tumors and cultured cells. Cell 1982;31:11–24. 6186379 10.1016/0092-8674(82)90400-7

[55] Chu PG, Weiss LM. Keratin expression in human tissues and neoplasms. Histopathology 2002;40:403–39. 12010363 10.1046/j.1365-2559.2002.01387.x

[56] Rhodes DR, Yu J, Shanker K, Deshpande N, Varambally R, Ghosh D, ONCOMINE: A Cancer Microarray Database and Integrated Data-Mining Platform. Neoplasia 2004;6:1–6. 15068665 10.1016/s1476-5586(04)80047-2 PMC1635162

[57] McGowan KM, Coulombe PA. Onset of keratin 17 expression coincides with the definition of major epithelial lineages during skin development. J Cell Biol 1998; 143:469–86. 9786956 10.1083/jcb.143.2.469 PMC2132846

[58] Mazzalupo S, Wong P, Martin P, Coulombe PA. Role for keratins 6 and 17 during wound closure in embryonic mouse skin. Dev Dyn 2003;226:356–65. 12557214 10.1002/dvdy.10245

[59] Coulombe PA, Tong X, Mazzalupo S, Wang Z, Wong P. Great promises yet to be fulfilled: defining keratin intermediate filament function in vivo. Eur J Cell Biol 2004;83:735–46. 15679118 10.1078/0171-9335-00443

[60] McNairn A, Guasch G. Epithelial transition zones: merging microenvironments, niches, and cellular transformation. Eur J Dermatol 2011;21:21–8. 21628126 10.1684/ejd.2011.1267

[61] Mitoyan L, Chevrier V, Hernandez-Vargas H, Ollivier A, Homayed Z, Pannequin J, A stem cell population at the anorectal junction maintains homeostasis and participates in tissue regeneration. Nat Commun 2021;12:2761. 33980830 10.1038/s41467-021-23034-x PMC8115161

[62] Schnabel J, Weber K, Hatzfeld M. Protein-protein interactions between keratin polypeptides expressed in the yeast two-hybrid system. Biochim Biophys Acta 1998;1403:158–68. 9630597 10.1016/s0167-4889(98)00036-6

[63] Snider NT, Omary MB. Post-translational modifications of intermediate filament proteins: mechanisms and functions. Nat Rev Mol Cell Biol 2014;15: 163–77. 24556839 10.1038/nrm3753 PMC4079540

[64] Yang L, Jin L, Ke Y, Fan X, Zhang T, Zhang C, E3 Ligase Trim21 Ubiquitylates and Stabilizes Keratin 17 to Induce STAT3 Activation in Psoriasis. J Invest Dermatol 2018;138:2568–77. 29859926 10.1016/j.jid.2018.05.016

[65] Hobbs RP, Jacob JT, Coulombe PA. Keratins Are Going Nuclear. Dev Cell 2016; 38:227–33. 27505414 10.1016/j.devcel.2016.07.022 PMC5511689

[66] Kumeta M, Hirai Y, Yoshimura SH, Horigome T, Takeyasu K. Antibody-based analysis reveals “filamentous vs. non-filamentous” and “cytoplasmic vs. nuclear” crosstalk of cytoskeletal proteins. Exp Cell Res 2013;319:3226–37. 23911988 10.1016/j.yexcr.2013.07.021

[67] Jacob JT, Nair RR, Poll BG, Pineda CM, Hobbs RP, Matunis MJ, Keratin 17 regulates nuclear morphology and chromatin organization. J Cell Sci 2020;133: jcs.254094. 10.1242/jcs.254094 PMC7648610 33008845

[68] Escobar-Hoyos L, Shah R, Roa-Peña L, Vanner E, Akalin A, Shroyer K. Keratin 17: cervical cancer prognostic marker promotes p27-nuclear export and tumor growth. FASEB J 2015;29:LB448. 10.1158/0008-5472.CAN-15-0293 26109559

[69] Turner JG, Dawson J, Sullivan DM. Nuclear export of proteins and drug resistance in cancer. Biochem Pharmacol 2012;83:1021–32. 22209898 10.1016/j.bcp.2011.12.016 PMC4521586

[70] Azmi AS, Khan HY, Muqbil I, Aboukameel A, Neggers JE, Daelemans D, Preclinical assessment with clinical validation of selinexor with gemcitabine and nab-paclitaxel for the treatment of pancreatic ductal adenocarcinoma. Clin Cancer Res 2020;26:1338–48. 31831564 10.1158/1078-0432.CCR-19-1728 PMC7073299

[71] Gorski JJ, James CR, Quinn JE, Stewart GE, Staunton KC, Buckley NE, BRCA1 transcriptionally regulates genes associated with the basal-like phenotype in breast cancer. Breast Cancer Res Treat 2010;122:721–31. 19882246 10.1007/s10549-009-0565-0

[72] Liao C, Xie G, Zhu L, Chen X, Li X, Lu H, p53 Is a direct transcriptional repressor of keratin 17: lessons from a rat model of radiation dermatitis. J Invest Dermatol 2016;136:680–9. 26747697 10.1016/j.jid.2015.12.021

[73] Shay JW, Wright WE. Role of telomeres and telomerase in cancer. Semin Cancer Biol 2011;21:349–53. 22015685 10.1016/j.semcancer.2011.10.001 PMC3370415

[74] Shay JW, Reddel RR, Wright WE. Cancer and Telomeres—An ALTernative to Telomerase. Science 2012;336:1388–90. 22700908 10.1126/science.1222394

[75] Telomerase activity in benign and malignant thyroid Tumors. Thyroid 1997;7: 337–42. 9226200 10.1089/thy.1997.7.337

[76] Nawaz S, Hashizumi TL, Markham NE, Laurie Shroyer A, Shroyer KR. Telomerase expression in human breast cancer with and without lymph node metastases. Am J Clin Pathol 1997;107:542–7. 9128266 10.1093/ajcp/107.5.542

[77] Bellacosa A, Kumar CC, Di Cristofano A. Activation of AKT kinases in cancer: implications for therapeutic targeting. Adv Cancer Res 2005;94:29–86. 16095999 10.1016/S0065-230X(05)94002-5

[78] Khanom R, Nguyen CTK, Kayamori K, Zhao X, Morita K, Miki Y, Keratin 17 is induced in oral cancer and facilitates tumor growth. PLoS One 2016;11: e0161163. 27512993 10.1371/journal.pone.0161163 PMC4981360

[79] Li C, Su H, Ruan C, Li X. Keratin 17 knockdown suppressed malignancy and cisplatin tolerance of bladder cancer cells, as well as the activation of AKT and ERK pathway. Folia Histochem Cytobiol 2021;59:40–48. 33577073 10.5603/FHC.a2021.0005

[80] Ruiz i Altaba A, Sánchez P, Dahmane N. Gli and hedgehog in cancer: tumours, embryos and stem cells. Nat Rev Cancer 2002;2:361–72. 12044012 10.1038/nrc796

[81] Callahan CA, Ofstad T, Horng L, Wang JK, Zhen HH, Coulombe PA, MIM/BEG4, a Sonic hedgehog-responsive gene that potentiates Gli-dependent transcription. Genes Dev 2004;18:2724–9. 15545630 10.1101/gad.1221804 PMC528890

[82] Yang H-Y, Wen Y-Y, Chen C-H, Lozano G, Lee M-H. 14–3–3 sigma positively regulates p53 and suppresses tumor growth. Mol Cell Biol 2003;23:7096–107. 14517281 10.1128/MCB.23.20.7096-7107.2003 PMC230310

[83] Enaka M, Nakanishi M, Muragaki Y. The gain-of-function mutation p53R248W suppresses cell proliferation and invasion of oral squamous cell carcinoma through the down-regulation of keratin 17. Am J Pathol 2021;191:555–66. 33307039 10.1016/j.ajpath.2020.11.011

[84] Takano M, Shimada K, Fujii T, Morita K, Takeda M, Nakajima Y, Keratin 19 as a key molecule in progression of human hepatocellular carcinomas through invasion and angiogenesis. BMC Cancer 2016;16:903. 27863477 10.1186/s12885-016-2949-y PMC5116168

[85] Smith-McCune K, Zhu YH, Hanahan D, Arbeit J. Cross-species comparison of angiogenesis during the premalignant stages of squamous carcinogenesis in the human cervix and K14-HPV16 transgenic mice. Cancer Res 1997;57:1294–300. 9102216

[86] Trenner A, Sartori AA. Harnessing DNA double-strand break repair for cancer treatment. Front Oncol 2019;9:1388. 31921645 10.3389/fonc.2019.01388 PMC6921965

[87] Tong X, Coulombe PA. Keratin 17 modulates hair follicle cycling in a TNFalpha-dependent fashion. Genes Dev 2006;20:1353–64. 16702408 10.1101/gad.1387406 PMC1472909

[88] Cave DD, Di Guida M, Costa V, Sevillano M, Ferrante L, Heeschen C, TGF-beta1 secreted by pancreatic stellate cells promotes stemness and tumourigenicity in pancreatic cancer cells through L1CAM downregulation. Oncogene 2020; 39:4271–85. 32291413 10.1038/s41388-020-1289-1 PMC7239770

[89] Zeng Y, Zou M, Liu Y, Que K, Wang Y, Liu C, Keratin 17 suppresses cell proliferation and epithelial-mesenchymal transition in pancreatic cancer. Front Med 2020;7:572494. 10.3389/fmed.2020.572494 PMC7726264 33324659

[90] Quinn JJ, Jones MG, Okimoto RA, Nanjo S, Chan MM, Yosef N, Single-cell lineages reveal the rates, routes, and drivers of metastasis in cancer xenografts. Science 2021;371:eabc1944. 33479121 10.1126/science.abc1944 PMC7983364

[91] Markey AC, Lane EB, Macdonald DM, Leigh IM. Keratin expression in basal cell carcinomas. Br J Dermatol 1992;126:154–60. 1371396 10.1111/j.1365-2133.1992.tb07813.x

[92] Wang W, Uberoi A, Spurgeon M, Gronski E, Majerciak V, Lobanov A, Stress keratin 17 enhances papillomavirus infection-induced disease by downregulating T cell recruitment. PLoS Pathog 2020;16:e1008206. 31968015 10.1371/journal.ppat.1008206 PMC6975545

[93] Lo BKK, Yu M, Zloty D, Cowan B, Shapiro J, McElwee KJ. CXCR3/ligands are significantly involved in the tumorigenesis of basal cell carcinomas. Am J Pathol 2010;176:2435–46. 20228225 10.2353/ajpath.2010.081059 PMC2861108

[94] Luengo A, Gui DY, Vander Heiden MG. Targeting metabolism for cancer therapy. Cell Chem Biol 2017;24:1161–80. 28938091 10.1016/j.chembiol.2017.08.028 PMC5744685

[95] Shukla SK, Purohit V, Mehla K, Gunda V, Chaika NV, Vernucci E, MUC1 and HIF-1alpha signaling crosstalk induces anabolic glucose metabolism to impart gemcitabine resistance to pancreatic cancer. Cancer Cell 2017;32:71–87. 28697344 10.1016/j.ccell.2017.06.004 PMC5533091

[96] Yan X, Yang C, Hu W, Chen T, Wang Q, Pan F, Knockdown of KRT17 decreases osteosarcoma cell proliferation and the Warburg effect via the AKT/mTOR/HIF1α pathway. Oncol Rep 2020;44:103–14. 32627037 10.3892/or.2020.7611 PMC7251737

