# Supplementary material for: Dissecting the Oncogenic Roles of Keratin 17 in the Hallmarks of Cancer
Source: Cancer Res. Author manuscript; Available in PMC 2022 Apr 19. (PMC9016724; doi:10.1158/0008-5472.CAN-21-2522)
Supplement: Supplmental Materials [file NIHMS1797836-supplement-Supplmental_Materials.pdf]

Supplemental Table 1: K17 is reported to be a diagnostic, prognostic, and predictive biomarker based on several publications. The table depicts which cancers K17 is published as a biomarker, along with the author and year of publication. K17 is published as a predictive biomarker in 1 cancer, prognostic biomarker in 13 cancers, and diagnostic biomarker in 10 cancers.

Diagnostic Biomarker		
**Cancer**	**Published by**	**Year**
Differentiated vulvar intraepithelial neoplasia	Dasgupta, Shatavisha *et al.*	2018
Cervical squamous cell carcinoma	Escobar-Hoyos, Luisa F et al.	2014
Basal cell carcinoma	Anderson-Dockter, Heidi *et al.*	2012
Urothelial carcinoma	Babu, Sruthi et al.	2019
Non-small cell lung cancer	Chen, Yuan *et al.*	2011
Oral squamous cell carcinoma	Kitamura, Ryoji *et al.*	2012
Larynx squamous cell carcinoma	Cohen-Kerem, Raanan *et al.*	2004
Pancreatic ductal adenocarcinoma	Lok, Terry *et al.*	2014
Colorectal Adenocarcinoma	Kim, Chan Yong *et al.*	2012
Anal squamous cell carcinoma	Nazarian, Rosalynn M *et al.*	2014
Prognostic Biomarker		
Oropharyngeal squamous cell carcinoma	Regenbogen, Elliot et al.	2018
Esophageal squamous cell carcinoma	Haye, Kester et al.	2021
Lung adenocarcinoma	Liu, Jianbo et al.	2018
Non-small cell lung cancer	Wang, Zhao et al.	2019
Epithelial ovarian carcinoma	Wang, Ya-Feng et al.	2013
Cervical squamous cell carcinoma	Escobar-Hoyos, Luisa F et al.	2014
Endometrial carcinoma	Bai, Ji Dong K et al.	2019
Endocervical adenocarcinoma	Mockler, Daniel et al.	2017
Gastric adenocarcinoma	Ide, Munenori et al.	2012
Pancreatic ductal adenocarcinoma	Roa-Pena, L., et al.	2019
Gallbladder adenocarcinoma	Kim, Kyungeun et al.	2017
Triple-negative breast cancer	Merkin, Ross D et al.	2017
Colorectal Cancer	Ujiie, Daisuke et al.	2020
Predictive Biomarker		
Pancreatic ductal adenocarcinoma	Roa-Pena, L., et al.	2019
